# Case Report: Orchiopexy in Two Poodle Dogs and Its Effect on Their Sperm Quality Parameters

**DOI:** 10.3389/fvets.2021.750019

**Published:** 2021-10-13

**Authors:** Feriel Yasmine Mahiddine, Min Jung Kim

**Affiliations:** Department of Research and Development, Mjbiogen Corp., Seoul, South Korea

**Keywords:** cryptorchidism, orchiopexy, dog, orchiectomy, sperm

## Abstract

Cryptorchidism is a common congenital abnormality encountered in veterinary clinics. The treatment of choice for this condition is a surgical procedure named orchiectomy or orchidectomy, where the retained testicle is removed. Surgical placement and fixation of the cryptorchid testicle into the scrotum, referred to as orchiopexy, is used in humans. However, due to the hereditary nature of cryptorchidism in dogs, this treatment option has not been proposed in veterinary clinics. Two adult Poodle dogs were referred to our research facility for a sperm parameter evaluation check. The two dogs were unilateral cryptorchid dogs treated with orchiopexy before the age of 6 months. Their sperm kinematics and morphology were within normal ranges, and their libido and testicles sizes were normal. Treatment of unilateral cryptorchidism by orchiopexy in dogs before the age of 6 months successfully restored spermatogenic function and sperm quality-related parameters. However, due to the nature of this condition, orchiectomy remains the treatment of choice.

## Introduction

Cryptorchidism is a congenital urological condition characterized by incomplete or absent testicular descent. In dogs, the testicles pass through the inguinal canal 3–4 days after birth and reach their final position in the scrotum on day 35 of life (1). Abnormal abdominal translocation and transinguinal migration result from incomplete or absent testicular descent due to abnormalities in the gubernaculum's outgrowth and regression or the persistence of cranial gonadal suspensory ligaments (1, 2). Different forms of cryptorchidism have been described based on whether one or both testicles are involved and where their position is (1, 3). Depending on the side, cryptorchidism can be bilateral or unilateral (1). The latter form is the most common (75% of the cases), with the right testicle being twice as likely to be retained (4, 5). Depending on the position, high abdominal, low abdominal, or inguinal cryptorchid testicles are the different forms that can be found (4, 6).

It is commonly encountered in small animal veterinary clinics, with an incidence of 1.2–12.9% in dogs (4, 5) and 1.7–3.8% in cats (4, 5). In dogs, it is an inherited, autosomal recessive trait, with a higher incidence reported in small breeds than in large breeds (5), especially in breeds such as Boxer, Cairn Terrier, Chihuahua, English Bulldog, Maltese, Miniature Poodle, Miniature Schnauze, Old English Sheepdog, Pekingese, Pomeranian, Shetland Sheepdog, and Toy Poodle (7). In general, a higher incidence of cryptorchidism in purebred dogs than in crossbred dogs has been reported (8).

The treatment of cryptorchidism by orchiopexy in dogs is a controversial topic, as cryptorchidism is a hereditary trait, and the risks of testicular neoplasia are still present in these animals (9). This surgery is no longer performed in veterinary hospitals, and cryptorchid dogs should not be included in reproductive programs. Although the treatment of choice for cryptorchidism is orchiectomy, we recently encountered two cases of orchiopexy performed in a private clinic. Kawakami et al. (10) reported that spermatogenesis was preserved after surgery in young dogs (10). However, no studies have been conducted on canine sperm morphology and kinematic parameters after orchiopexy. In this case report, we evaluated the morphological and kinematic parameters of sperm from two dogs who had undergone orchiopexy surgery.

## Case Presentation

Two unilateral cryptorchidic Toy Poodle dogs that underwent orchiopexy were referred to our research facility (Mjbiogen) in Seoul, South Korea, for the evaluation of their sperm kinematic and morphological parameters. Orchiopexy was performed at 2 months of age in a private clinic 2 years ago. No information about the surgical procedure or the location of the cryptorchid testicles was disclosed, and the owners gave their approval for the use of these results for publication. After reaching puberty, the owners reported successful pregnancies and delivery of healthy offsprings, using semen from these dogs. To assess the effects of the surgery on sperm quality, we collected semen from these dogs (*n* = 2), analyzed the sperm quality parameters, and compared them with those of normal, age-matched toy poodles (*n* = 3) from the same owner. Statistical analysis was performed using GraphPad Prism 5.0 (GraphPad, CA, San Diego, USA), and data were analyzed using one-way analysis of variance (ANOVA) following by a Tukey's multiple comparison test. All dogs were fed commercial adult dry food, and water was provided ad libitum. Semen was obtained by digital manipulation in the presence of a bitch in estrus, and only the second fraction of the ejaculate was collected and processed for evaluation in the laboratory. During collection, libido in the operated dogs was not diminished nor lower than that of the control dogs. To assess any difference in the testicles sizes, the lengths of the longitudinal and transverse axes of the right and left testicles were measured using a simple measuring flat ruler. The results for each testicle were transcribed into a table, and the orchiopexy and normal testicles were compared (Table 1). At palpation, the operated testicle had no deformation, but in one of the dogs the right testicle was slightly smaller than the left testicle (Orchiopexy dog 1).

**Table 1:** Lengths of the longitudinal and transverse axes of the right and left testicles in orchiopexy and control dogs.

**Parameters**	**Orchiopexy dog 1**	**Orchiopexy dog 2**	**Control dogs**
Right longitudinal axis (cm)	1.9	2	2.4 ± 0.1
Left longitudinal axis (cm)	2.4	3	2.7 ± 0.1
Right transverse axis (cm)	1.1	1.3	1.4 ± 0.1
Left transverse axis (cm)	1.3	1.2	1.5 ± 0.1

Each collected semen sample (*n* = 3) was diluted with Tris-extender (1:1 [v/v]—distilled water, Tris (hydroxymethyl) aminomethane 24 g/L, citric acid 14 g/L, fructose 8 g/L, and kanamycin sulfate 0.15 g/L; pH 6.6, 290 mOsm) and centrifuged at 700 × *g* for 1 min. The supernatant was collected and centrifuged (500 × *g*/5 min), and only the pellet was resuspended in Tris-extender to achieve a concentration of 200 × 10^6^ sperm/ml. Transportation media−54% (v/v) Tris-extender, 40% (v/v) egg yolk, and 6% (v/v) glycerol—was added, and the samples were stored at 4°C to be transported to the laboratory, 1 h away from the breeding facility. Once in the laboratory, each sample was washed and resuspended in Tris-extender before proceeding for further analysis. The sperm quality-related parameters assessed were motility and kinematic parameters, viability, and morphology parameters, using a computer-assisted sperm analysis (CASA; Sperm Class Analyzer® System version 6.4.0.93, Microptic, Barcelona, Spain). The system included a Nikon Eclipse ci-L microscope (Nikon, Tokyo, Japan) with a ×10 phase-contrast objective. Leja 20-μm chamber slides (Leja, Gynotec Malden, Nieuw Vennep, the Netherlands) were used for the analysis, and the frame rate was set at 25 frames/s. Sperm motility, progressive motility, curvilinear velocity (VCL), straight-line velocity (VSL), average path velocity (VAP), linearity (LIN), straightness (VSL/VAP) (STR), wobble VAP/VCL (WOB), the amplitude of lateral head, and beat cross frequency were analyzed (Table 2). No differences were observed between the orchiopexy dogs and the control dogs, indicating that dogs that underwent orchiopexy surgery have sperm kinematic parameters within the normal ranges.

**Table 2:** Kinematic parameters, live cell, and intact acrosome percentages in orchiopexy and control dogs.

**Parameters**	**Orchiopexy dog 1**	**Orchiopexy dog 2**	**Control dogs**
Concentration (M/ml)	355.1 ± 167.7	577.1 ± 51.9	592.6 ± 71.9
Semen volume (ml)	1.8 ± 0.4	1.5 ± 0.1	0.7 ± 0.1
Motility (%)	89.7 ± 5.3	99.1 ± 0.5	96.8 ± 1.4
Progressive motility (%)	49.8 ± 9.8	61.4 ± 2.4	54.6 ± 6.8
VCL (μm/s)	80.5 ± 12.9	119.8 ± 3.5	103.0 ± 9.6
VAP (μm/s)	49.6 ± 6.5	64.5 ± 1.4	58.9 ± 5.5
VSL (μm/s)	34.7 ± 3.0	39.6 ± 2.4	37.1 ± 3.5
LIN (%)	42.2 ± 4.7	31.8 ± 1.1	34.8 ± 1.9
STR (%)	66.2 ± 5.6	57.6 ± 1.9	59.0 ± 2.6
WOB (%)	61.2 ± 1.9	53.7 ± 0.5	57.2 ± 0.9
ALH (μm)	2.1 ± 0.4	3.1 ± 0.1	2.7 ± 0.2
BCF (Hz)	10.4 ± 1.1	10.5 ± 1.0	10.8 ± 0.8
Live cell percentage (%)	75.3 ± 8.0	76.8 ± 2.5	72.9 ± 3.8
Intact acrosome (%)	89.7 ± 4.4	93.5 ± 3.0	94.6 ± 1.1

*VCL, curvilinear velocity; VAP, average path velocity; VSL, straight-line velocity; LIN, linearity; STR, straightness (VSL/VAP); WOB, wobble (VAP/VCL); ALH, amplitude of lateral head displacement; BCF, beat cross frequency*.

**Figure 1:**
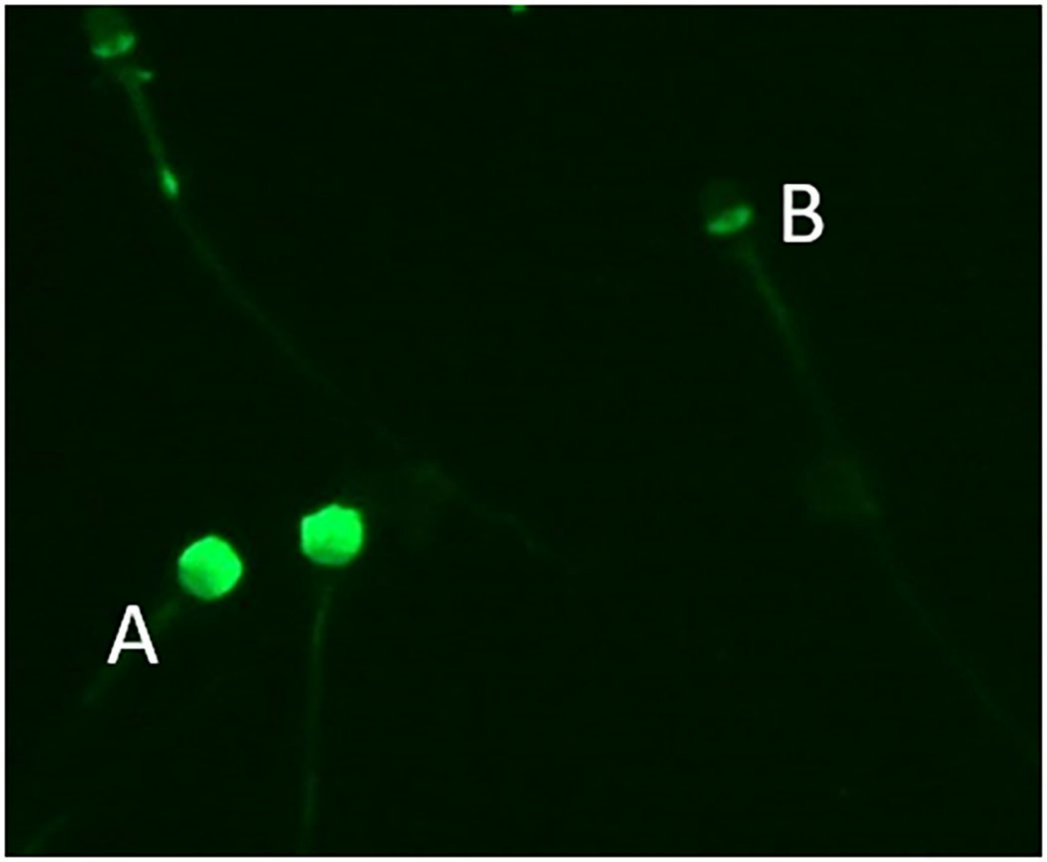
Sperm acrosome stained with fluorescein isothiocyanate-conjugated peanut agglutinin (FITC-PNA). Sperm with **(A)** intact acrosome and **(B)** non-intact acrosome.

Eosin Y staining was used to determine the percentage of live sperm cells. In brief, the samples were washed, and a drop of 10 μ**l** from the sperm pellet with an equal amount of 0.5% eosin Y solution was mixed and smeared onto warm glass slides. The slides were then air-dried, and the sperm were evaluated. For each stained smear, 200 sperm were examined with a light microscope (Eclipse Ts 2, Nikon, Tokyo, Japan) with an oil immersion objective lens (×1000 magnification) (11). The unstained sperm were counted as alive, and the stained sperm were counted as dead cells. The results are expressed as the percentage of live sperm cells (12) (Table 2) and showed no difference between orchiopexy dogs and control dogs. The sperm acrosome membrane was analyzed using fluorescein isothiocyanate-conjugated peanut agglutinin (FITC-PNA), as previously described (13). In brief, semen was smeared on glass slides, air-dried, fixed in absolute methanol, stained, and mounted with anti-fade mounting medium (VECTASHIELD®, Vector Laboratories, Burlingame, CA, USA). The integrity of the sperm acrosome membrane was analyzed using an epifluorescence phase-contrast microscope (Eclipse Ts 2, Nikon, Tokyo, Japan) and classified as intact acrosome (strong green fluorescence) or non-intact acrosome (partial or no fluorescence) (14) (Table 2 and Figure 1). Our results showed that acrosome integrity was preserved in orchiopexy dogs. Diff-Quick staining was used to assess morphological defects. Each sample was washed and smeared onto warm glass slides, fixed in methanol, and stained with eosin as an anionic/acidic dye and with methylene blue as a cationic dye. The slides were air-dried, and morphology was assessed using a light microscope (Eclipse Ts 2, Nikon, Tokyo, Japan) with an oil immersion objective lens (×1,000 magnification). Head defects, droplet, coiled, and bent tail defects were evaluated in each sample (15) (Table 3). Morphology parameters were similar in orchiopexy and control dogs, which shows that orchiopexy at a young age in dogs has no negative effects on sperm morphology and that spermiogenesis is preserved.

**Table 3:** Percentage of morphological defects in orchiopexy and control dogs.

**Parameter**	**Orchiopexy dog 1**	**Orchiopexy dog 2**	**Control dogs**
Morphological defects	2.5 ± 1.3	1.0 ± 0.3	2.2 ± 1.0

## Discussion

An orchiopexy is the surgical fixation of testicles in the scrotum (16). It can be performed through open surgery techniques or laparoscopy (17). The incidence of undescended testis (UDT) in humans is 1% in male infants, and it is considered as one of the most common congenital abnormalities in males (18). However, unlike cryptorchidism in dogs, UDT is corrected with orchiopexy. The hereditary character of cryptorchidism in dogs makes it hard, and almost unethical, to perform this surgery on cryptorchid males (8, 19). Moreover, cryptorchidism can be associated with other congenital abnormalities (8, 20), and the risks of developing a neoplasia in testicles subjected to orchiopexy are higher (6, 9). The aim of this case study is to assess the sperm parameters in two cryptorchid dogs; however, for all the aforementioned reasons, orchiopexy should not be suggested by practicians to cryptorchid dogs owners.

In humans, early orchiopexy (<1 year old) was associated with significantly higher sperm count and motile sperm in comparison with cases where orchiopexy was performed later (1–2 years of age) (21). As orchiopexy is a controversial surgery in veterinary medicine, the ideal age to perform this surgery has never been established. In dogs, the final diagnosis of cryptorchidism can only be made after 6 months of age (22). However, since testicles are expected to reach their final position around day 35 of life (23), cryptorchidism can be suspected in pups aged ≥2 months.

In this case, according to the owners, orchiopexy was performed on the two Poodle dogs at 2 months of age to ensure a higher percentage of full recovery of spermatogenetic function. When the testicles stay in the abdominal cavity, where the temperature is higher, spermatogenesis is compromised. Since testicles' sensitivity to temperature is high, a surgery performed later could result in the absence of spermatogenesis or the presence of poor quality sperm with low fertilizing ability (24). From our own data, a beagle dog with unilateral cryptorchidism showed motility and progressive motility parameters of 24.7 ± 29.4% and 29.8 ± 22.3%, respectively (unpublished data), which shows that the sperm kinematic parameters of unilateral cryptorchid dogs are lower than those of normal dogs.

In humans, males who underwent unilateral orchiopexy at a young age (before 8 years of age) have a good prognosis for fertility compared to males who were operated on later or for both testicles (25). In this case, dogs who underwent unilateral orchiopexy before the age of 6 months had sperm kinematic and morphological parameters within the normal range. It should be noted that Orchiopexy dog 2 sperm parameters, especially sperm total motility and progressive motility, were slightly higher than those of the other dogs but were not statistically significant (*p*-value > 0.05). Morphological defects in Orchiopexy dog 2 sperm samples were also lower than those of the control dogs and Orchiopexy dog 1 (Table 3). In addition, Orchiopexy dog 1 sperm concentration and acrosome integrity were lower than those of control dogs and Orchiopexy dog 2, without statistical significance (p-value > 0.05) (Table 2). This difference in sperm concentration between Orchiopexy dog 1 and the other dogs could also be attributed to the difference in testicular sizes (Table 1) (26). From these results, unilateral cryptorchid dogs treated with early orchiopexy may have a good prognosis for fertility, and their sperm quality parameters are similar to those of fertile control dogs.

## Conclusion

Here, we report two cases of dog orchiopexy in which the sperm kinematic and morphological parameters were similar to those from healthy age-matched dogs of the same breed. Like in humans, orchiopexy at a young age in dogs seems to preserve sperm quality parameters as our results show that orchiopexy before the age of 6 months did not alter sperm quality parameters in these two dogs. This makes dogs as one potential orchiopexy study model for humans. However, this remains a controversial surgery due to the hereditary aspect of cryptorchidism and the risk of testicular neoplasia. The results from this case study should not encourage dog owners or practitioners to perform it. Therefore, we recommend veterinarians to warn owners about this surgery and its dangers and dissuade them from considering it as a treatment option.

## Data Availability Statement

The original contributions presented in the study are included in the article/supplementary material, further inquiries can be directed to the corresponding author/s.

## Ethics Statement

Ethical review and approval was not required for the animal study because it is a clinical case and the owners gave their permission. Written informed consent was obtained from the owners for the participation of their animals in this study.

## Author Contributions

MK participated in collecting owners approval, semen samples, and revising the manuscript. FM participated in collecting semen samples and results and drafting and revising the manuscript. Both authors contributed to the article and approved the submitted version.

## Funding

Cooperative research program of Rural Development Administration (#PJ014786012021).

## Conflict of Interest

The authors were employed by company Mjbiogen Corp.

## Publisher's Note

All claims expressed in this article are solely those of the authors and do not necessarily represent those of their affiliated organizations, or those of the publisher, the editors and the reviewers. Any product that may be evaluated in this article, or claim that may be made by its manufacturer, is not guaranteed or endorsed by the publisher.
